# An emotion recognition subtyping approach to studying the heterogeneity and comorbidity of autism spectrum disorders and attention-deficit/hyperactivity disorder

**DOI:** 10.1186/s11689-018-9249-6

**Published:** 2018-11-15

**Authors:** Francesca Waddington, Catharina Hartman, Yvette de Bruijn, Martijn Lappenschaar, Anoek Oerlemans, Jan Buitelaar, Barbara Franke, Nanda Rommelse

**Affiliations:** 1 Department of Human Genetics, Radboud University Medical Center Nijmegen, Geert Grooteplein Zuid 10, 6525 GA Nijmegen, The Netherlands; 2 Donders Institute for Brain, Cognition and Behaviour, Radboud University, Nijmegen, The Netherlands; 3 Department of Psychiatry, Interdisciplinary Center Psychopathology and Emotion Regulation (ICPE), University Medical Center Groningen, University of Groningen, Groningen, The Netherlands; 4 Karakter Child and Adolescent Psychiatry University Centre, Reinier Postlaan 12, 6525 GC Nijmegen, The Netherlands; 5 Department of Geriatrics, Radboud University Medical Centre, Nijmegen, The Netherlands; 6 Department of Psychiatry, Radboud University Medical Centre, Nijmegen, The Netherlands; 7 Department of Cognitive Neuroscience, Radboud University Medical Centre, Nijmegen, The Netherlands

**Keywords:** Factor mixture modelling, Autism spectrum disorders, Attention-deficit/hyperactivity disorder, Emotion recognition, Latent class

## Abstract

**Background:**

Emotion recognition dysfunction has been reported in both autism spectrum disorders (ASD) and attention-deficit/hyperactivity disorder (ADHD). This suggests that emotion recognition is a cross-disorder trait that may be utilised to understand the heterogeneous psychopathology of ASD and ADHD. We aimed to identify emotion recognition subtypes and to examine their relation with quantitative and diagnostic measures of ASD and ADHD to gain further insight into disorder comorbidity and heterogeneity.

**Methods:**

Factor mixture modelling was used on speed and accuracy measures of auditory and visual emotion recognition tasks. These were administered to children and adolescents with ASD (*N* = 89), comorbid ASD + ADHD (*N* = 64), their unaffected siblings (*N* = 122), ADHD (*N* = 111), their unaffected siblings (*N* = 69), and controls (*N* = 220). Identified classes were compared on diagnostic and quantitative symptom measures.

**Results:**

A four-class solution was revealed, with the following emotion recognition abilities: (1) average visual, impulsive auditory; (2) average-strong visual and auditory; (3) impulsive/imprecise visual, average auditory; (4) weak visual and auditory. The weakest performing class (4) contained the highest percentage of patients (66.07%) and the lowest percentage controls (10.09%), scoring the highest on ASD/ADHD measures. The best performing class (2) demonstrated the opposite: 48.98% patients, 15.26% controls with relatively low scores on ASD/ADHD measures.

**Conclusions:**

Subgroups of youths can be identified that differ both in quantitative and qualitative aspects of emotion recognition abilities. Weak emotion recognition abilities across sensory domains are linked to an increased risk for ASD as well as ADHD, although emotion recognition impairments alone are neither necessary nor sufficient parts of these disorders.

**Electronic supplementary material:**

The online version of this article (10.1186/s11689-018-9249-6) contains supplementary material, which is available to authorized users.

## Background

Autism spectrum disorder (ASD) and attention-deficit/hyperactivity disorder (ADHD) are highly comorbid, which is likely due to shared aetiological mechanisms [1, 2]. Although many models of the relationship between ASD, ADHD, and their comorbidity have been hypothesised [2, 3], the inherent heterogeneity within both diagnostic categories complicates the study of the relationship between these disorders and their developmental trajectories [4, 5]. Overlap has also been noted in the cognitive deficits found in these disorders, including emotion recognition dysfunction. This important aspect of social cognition has been reported as being impaired in many disorders including ASD, ADHD, and disorders they are comorbid with, such as depression and oppositional defiant disorder (for a review see [6]).

For ASD, there is already a considerable body of literature regarding emotion recognition, for visual emotion recognition abilities in particular, while this area of research is relatively underdeveloped in ADHD. Nonetheless, studies of ASD and also ADHD have demonstrated visual emotion recognition impairments for specific emotions [7–17] and impairments for visually identifying all emotions [18–29]. However, other studies did not report impairments in recognising emotions at all [30–33]. Indeed, meta-analyses speak to the heterogeneity of visual emotion recognition performance in both ASD and ADHD [34–36].

For auditory emotion recognition, the literature is less advanced for both ASD and ADHD. Similar to visual emotion recognition, this literature also demonstrates inconsistent results, with auditory emotion recognition in both ASD and ADHD suggested to be comparable to controls [10, 17, 22, 24, 32, 37–39], worse than controls [19, 23, 24, 40–42], or only worse for specific emotions [12, 43–45].

Comparisons of emotion recognition impairments in ASD and ADHD are scarce. Bora and Pantellis [34] indirectly compared visual emotion recognition in ASD and ADHD and suggested that impairments were present in ADHD, although potentially milder than ASD. Only a handful of studies directly compared ASD and ADHD and occasionally comorbid ASD + ADHD [8, 25, 40, 46–48]. Similar to the findings for the disorders individually, the results of the comparisons were also inconsistent, with ASD and ADHD not demonstrating significant differences in emotion recognition impairments [46, 48] or showing quantitative differences [40]. Berggren et al. [8] and Van der Meer et al. [47] found overall differences in visual emotion recognition reaction time, but not in accuracy between ASD (+ ADHD) and ADHD. Therefore, to what extent emotion recognition impairments are similar and/or unique in ASD and ADHD is still largely unknown due to these heterogeneous results.

Previous research has demonstrated that empirical subtyping is a valuable tool for improving our understanding of ASD and ADHD [4, 5, 49–51]. Techniques that explicitly utilise heterogeneity, as observed for emotion recognition abilities in ASD and ADHD, can be used to optimally investigate overlap and differences between disorders. In our previous study [48], we identified a factor structure of emotion recognition that underlies ASD and ADHD, which can serve as a useful basis to identify homogeneous subgroups. Factor mixture modelling (FMM) can be used for this and is a logical extension of factor analysis, as it combines factor analysis with latent class analysis. It therefore allows the data to be viewed from categorical, person-based perspectives, and dimensional, variable-based perspectives simultaneously. This modelling technique can identify homogeneous subgroups without a-priori specifications, the use ofclass probabilities allows for classes with subtle differences to be identified, statistics determining the goodness-of-fit can be used to determine the most appropriate model and independence of participants is not an issue because family structures can be taken into account. In this way, FMM has advantages over other clustering methods, such as hierarchical clustering and community detection [52–54].

Consequently, the current study aimed to investigate the heterogeneity of emotion recognition in individuals with neurodevelopmental disorders ASD and ADHD, and its potential as a transdiagnostic phenotype by identifying emotion recognition subtypes with FMM. The emotion recognition data was reduced to underlying, continuously distributed factors that drive performance on emotion recognition tasks (as identified in Waddington et al. [48]). These factors were then used to create homogeneous subgroups of participants that have very similar emotion recognition profiles. By including patients with ASD, ADHD, and ASD + ADHD, unaffected siblings of ASD and ADHD patients, and healthy controls, we covered the entire spectrum from low risk (controls) to moderate risk (unaffected siblings) and high risk (patients) for emotion recognition dysfunction. The FMM-based emotion recognition subgroups were compared on their diagnostic (ASD, ADHD) and quantitative symptom profiles (autism symptom severity, inattention, hyperactivity/impulsivity, oppositional behaviour, anxiety) to examine how emotion recognition dysfunction relates to (cross-disorder) symptoms. To our knowledge, this is the first study to directly compare pure and comorbid ASD and ADHD on emotion recognition deficits and the role of ASD and ADHD behavioural symptoms, whilst accounting for heterogeneity.

## Methods

### Participants

The data used in this study originally came from two cohorts, the NeuroIMAGE study, which is a follow-up (2009–2012) of the Dutch part of the International Multicenter ADHD Genetics (IMAGE) study performed between 2003 and 2006 [55–58] and the Biological Origins of Autism (BOA) study [59]. In these cohort studies, the recruited patient families were included if (1) they had one child with a clinical diagnosis of ADHD (NeuroIMAGE) or ASD (BOA) and (2) at least one biological sibling (regardless of possible clinical diagnosis) willing to participate. Healthy control families had at least one child, with no formal or suspected ADHD or ASD in the participant or any first-degree relatives. All participants were of European Caucasian descent. In both cohorts, the exclusion criteria were an IQ < 70, a diagnosis of epilepsy, brain disorders, known genetic disorders (e.g. down syndrome or fragile X syndrome), with an additional criterion of a clinical diagnosis of autistic disorder or Asperger disorder in the NeuroIMAGE cohort. For the current study, a subsample of participants was selected from both cohorts, which was matched on mean chronological age (M = 12.6 years, SD = 2.4, age range 7–18 years; see Additional file 1: Figure S1 and Table S4 for details). The number of comorbid ASD + ADHD unaffected siblings was relatively small, and subsequently, these unaffected siblings were grouped with the ASD-unaffected siblings. In total, 89 participants with ASD (further mentioned as ASD-only patients), 64 participants with comorbid ASD + ADHD (further mentioned as ASD + ADHD patients), 122 of their unaffected siblings, 111 patients with ADHD (further mentioned as ADHD-only patients), 69 of their unaffected siblings, and 220 healthy controls were included. The BOA data used in this study partly overlaps (79%) with a previous study [12].

### Diagnostic assessment

All participants were phenotyped for ASD and ADHD using validated and standardised questionnaires and diagnostic interviews. Briefly, children already clinically diagnosed with ASD and/or ADHD, their siblings, and the control children were screened for the presence of ASD and ADHD symptoms using the parent-reported Social Communication Questionnaire (SCQ) [60] and the parent- and teacher-reported Conners Rating Scales—Revised (CPRS and CTRS respectively) [61]. Raw scores of ≥ 10 on the SCQ total score and *T* scores ≥ 63 on the Conners DSM-IV inattention, hyperactivity-impulsivity, or combined scales were considered as potential clinical cases.

All youth scoring above cutoff on any of the screening questionnaires underwent full clinical ADHD assessment using the Parental Account of Childhood Symptoms ADHD subversion (PACS) for ADHD (BOA cohort) [62] or the Schedule for Affective Disorders and Schizophrenia for School-Age Children—Present and Lifetime Version (K-SADS; in NeuroIMAGE) [63]. Clinical assessment for ASD was performed using the Autism Diagnostic Interview-Revised (ADI—R) structured interview for ASD (BOA cohort) [64]. Control youth were required to obtain non-clinical scores (i.e. a raw score < 10 on the SCQ and *T* score < 63 on both CPRS and CTRS) to qualify for this study (further details in Additional file 1).

### Cognitive measures

#### Emotion recognition

Speed (mean reaction time) and accuracy (percentage of errors) of visual and auditory emotion recognition were measured using the Identification of Facial Emotions (IFE) task and the Affective Prosody (AP) task from the battery of the Amsterdam Neuropsychological Tasks (ANT) [65]. In the IFE task, participants viewed individual photos of facial expressions and indicated if they saw the target emotion (happy, fearful, or angry) in these photos (Additional file 1: Figure S1). In the AP task, participants listened to sentences of neutral content that differed in prosody. The participants had to identify the emotion (happy, fearful, sad, or angry) of the voice they heard. Both tasks are fully described elsewhere [12].

#### Intelligence

An estimate of the Full-Scale Intelligence Quotient (FSIQ) was derived from two subtests (Vocabulary and Block Design) of the Wechsler Intelligence Scale for Children version III (WISC-III) [66] for participants younger than 16 years or the Wechsler Adult Intelligence Scale version III (WAIS-III) [67] for participants 16 years and older.

### Procedure

The tasks described were part of broader assessment batteries used in the BOA and NeuroIMAGE cohorts. Testing was conducted in quiet rooms with minimal distractions. Participants were asked to withhold use of psychoactive drugs for at least 24 h before measurement. During the testing day, participants were motivated with short breaks, and at the end of the day, the children were rewarded. Both studies were approved by the local medical ethics board. Written informed consent was obtained from all participants and their parents (parents signed informed consent for participants under 12 years of age).

### Analyses

SPSS version 22 was used for the analysis of the data. Less than 5% of the data was missing. Data was imputed for each cohort separately using SPSS based on the data from the IFE and AP tasks as well as gender, age, IQ, and family and diagnostic status. The measures for both cohorts together were normalised and standardised using Van der Waerden transformation, and the IQ scoring was reversed. Consequently, all of the variables had scores on the same *z* scale, with lower scores implying better performance (fewer errors, faster reaction times, and a higher IQ). Standardised age-regressed residuals were calculated.

The model-fitting analyses were performed using MPlus version 6 [68]. The stepwise FMM strategy suggested by [52] was used on the combined cohorts, using the models derived from less demanding techniques (i.e. confirmatory factor analysis (CFA), latent class analysis (LCA)) as input for the more complex FMM estimation process. The following four factors underlying the 14 emotion recognition variables from the previous factor analysis of this sample [48] were used: visual speed, visual accuracy, auditory speed, and auditory accuracy. As the next step, LCA was conducted on the four factors to explore how many classes could be distinguished. Subsequently, we conducted the FMM, using the models derived from CFA conducted in Waddington et al. [48] and LCA to guide model fitting. For all stages of the LCA and FMM, the model fit was assessed using the Akaike information criteria (AIC), Bayesian information criteria (BIC), Lo-Mendell-Rubin test, and entropy. The details of criterion for adequate fit and the results of the models can be found in the Additional file 1: Tables S1–S3.

Mixed models were used to assess the differences between the classes on scores for the emotion recognition factors, speed-accuracy trade-offs in the visual and auditory domains, as well as gender, age, IQ, and phenotypic measures. Additionally, chi-square tests were performed to assess differences in diagnostic proportions between the classes. Multiple comparisons were corrected according to the false discovery rate (FDR) controlling procedure, with the *q* value set at .05. Post hoc analyses comparing patients and controls separately across classes were conducted to assess if different emotion recognition subtypes within diagnostic groups were related to symptoms. A comprehensive overview of the results of the mixed models and *T* tests can be found in the Additional file 1: Tables S5–S6.

## Results

**Fig. 1:**
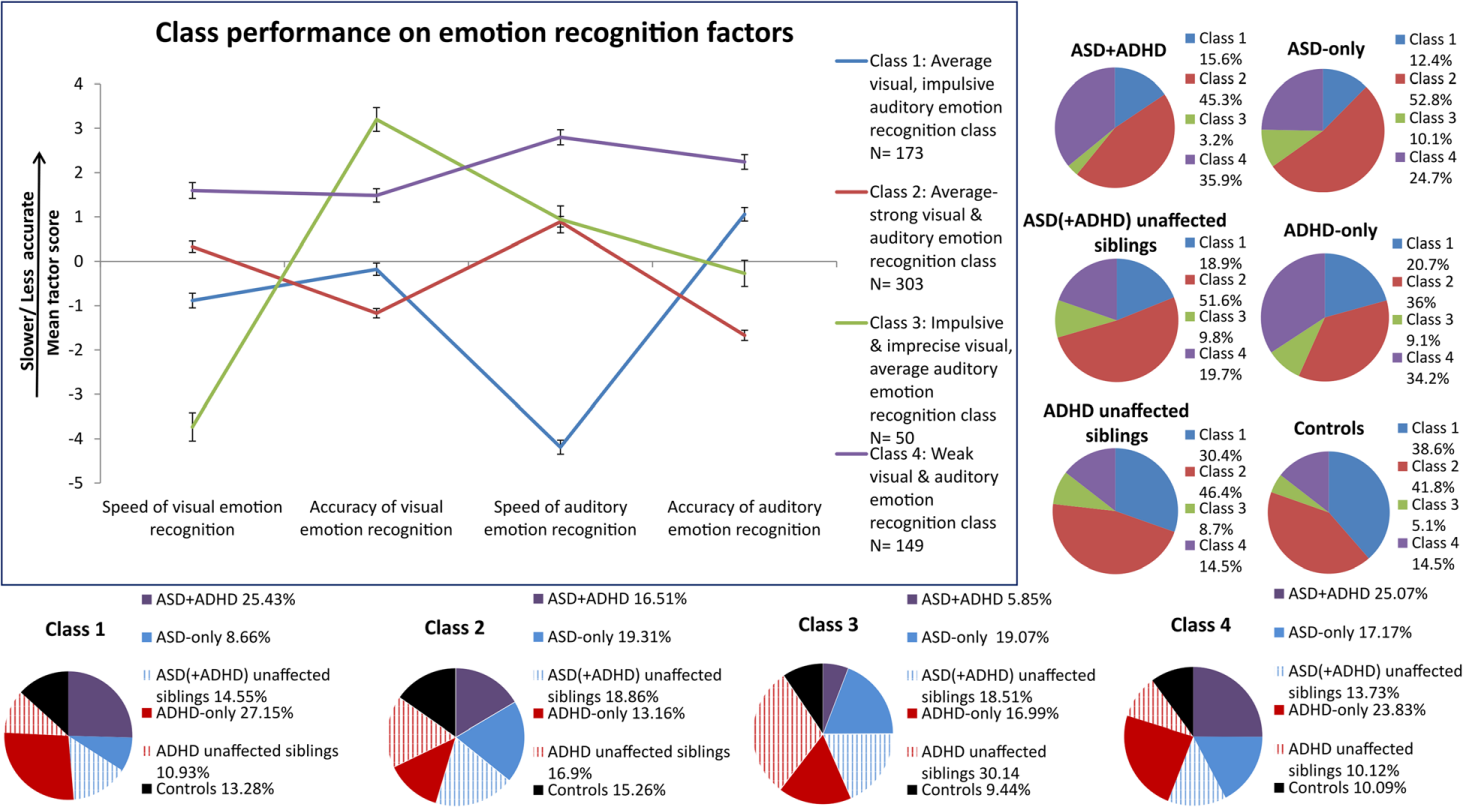
Box: emotion recognition classes identified in the current study across patients with pure and comorbid ASD and ADHD, their unaffected siblings, and controls. Each line represents the emotion recognition profile (mean factor scores − speed and accuracy of IFE and AP tasks ± 1 S.E.) for each class. Lower scores represent faster reaction time and fewer errors made. An age covariate was applied. Lower panel: pie charts represent the within-class weighted proportions of each diagnostic group. For each class, diagnostic groups were weighted using a weighting coefficient of %within diagnostic group/% within-class. Right panel: pie charts represent the proportion of each diagnostic group across each class

Utilising the four-factor solution (visual speed, visual accuracy, auditory speed, and auditory accuracy) identified in Waddington et al. [48] for this sample, four classes were suggested in the LCA. A four-factor four-class solution was therefore used as an initial criterion to guide the fitting of the FMM, which had an optimal fit for the data (see Additional file 1 for details). The classes were labelled according to the characteristics of their emotion recognition profiles (Fig. 1, Additional file 1: Tables S4–S6). Class 4 (*n* = 149) was the worst-performing class, demonstrating weaknesses on both visual and auditory emotion recognition. This class was significantly slower and less accurate than the other classes across visual and auditory emotion recognition, with the exception of class 3 on visual accuracy. Comparing modalities within class 4, participants were significantly faster and more accurate at visual than auditory emotion recognition (visual versus auditory speed: *p*_FDR_ < .001, *d* = 0.77; visual versus auditory accuracy: *p*_FDR_ = .004, *d* = 0.48). Class 2 (*n* = 303) was an average-performing class, having average to strong visual and auditory emotion recognition abilities, relative to the other classes. This class was faster and more accurate than class 4 in both visual and auditory emotion recognition. Class 2 also showed signs of a speed-accuracy trade-off, favouring accuracy over speed (visual speed versus visual accuracy: *p*_FDR_ < .001, *d* = 0.98; auditory speed versus auditory accuracy: *p*_FDR_ < .001, *d* = 2.00). Classes 1 and 3 can be referred to as an impulsive auditory, average visual emotion recognition class and an impulsive and imprecise visual, average auditory emotion recognition class, respectively. Class 1 (*n* = 173) showed significant speed-accuracy trade-offs, trading both visual and auditory accuracy in favour of speed, strongest for auditory recognition (visual speed versus visual accuracy: *p*_FDR_ = .001, *d* = 0.51; auditory speed versus auditory accuracy: *p*_FDR_ < .001, *d* = 5.84). Class 3 had a greater speed-accuracy trade-off in visual emotion recognition: *p*_FDR_ < .001, *d* = 13.87) than auditory emotion recognition: *p*_FDR_ = .003, *d* = 0.88). The inclusion of age, sex, and IQ covariates did not attenuate these effects.

The four identified classes differed in IQ (*F*(3, 671) = 15.17, *p*_FDR_ *< .001*, *d* = 0.24) and sex (*F*(3, 671) = 5.33, *p*_FDR_ *= .002*, *d* = − 0.30), but not in age (*F*(3, 671) = 1.83, *p*_FDR_ = *.14 d* = − 0.05). Class 4 had a significantly lower IQ than the other classes (class 4 versus class 1: *p*_FDR_ < .001, *d* = 0.43; class 4 versus class 2: *p*_FDR_ < .001, *d* = 0.40; class 4 versus class 3: *p*_FDR_ = .006, *d* = 0.45). The proportion of males did not differ significantly between the classes (see Additional file 1: Table S7).

Sex and IQ affected the individual factors differentially. Sex and IQ only affected accuracy factors (*visual*—sex: *F*(1, 675) = 7.27, *p*_FDR_ = .028, *d* = 0.02; IQ: *F*(1, 675) = 5.14, *p*_FDR_ = .048, *d* = 0.02; *auditory*—sex: *F*(1, 675) = 9.20, *p*_FDR_ = .006, *d* = − 0.21; IQ: *F*(1, 675) = 28.19, *p*_FDR_ = .006, *d* = − 0.36), but not visual or auditory speed.

## Behavioural and diagnostic profiles of the classes

**Fig. 2:**
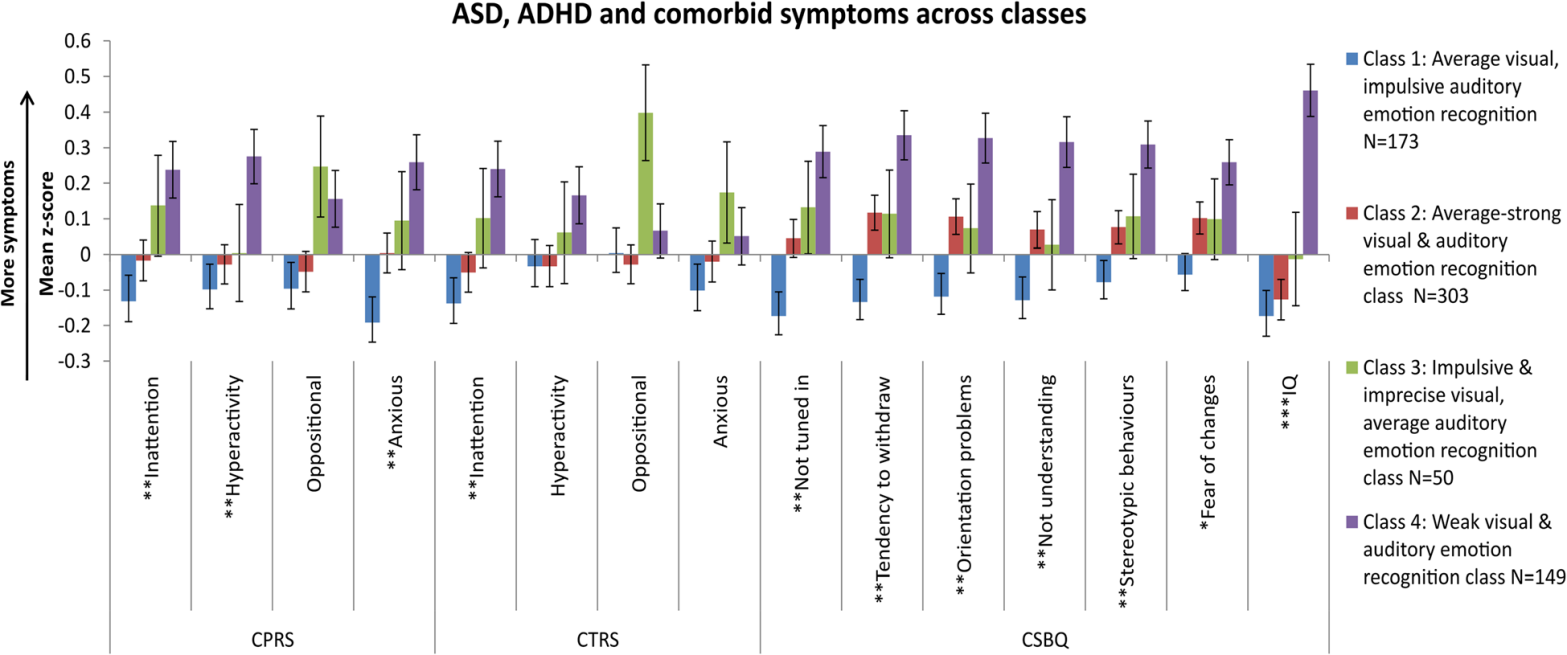
Class profiles of IQ and ASD, ADHD, and comorbid symptoms. Each bar represents the mean *z* score (± 1 S.E.) for ADHD symptoms from Conners’ Parents Rating Scale (CPRS), Conners’ Teachers Ratings Scale (CTRS), and the Children’s Social Behaviour Questionnaire (CSBQ). IQ *z* scores were reversed (higher IQ *z* score = lower IQ). One asterisk indicates the significant difference between classes 1 and 4. Two asterisks indicate the significant differences between classes 1 and 4 or classes 2 and 4. Three asterisks indicate the significant differences between class 4 and classes 1, 2, and 3

As shown in Fig. 2 and Additional file 1: Table S7, when testing dimensional measures of ASD- and ADHD-related behaviour, class 1 had lowest symptom levels for ASD, ADHD, and related behaviours, whereas class 4 had the highest. These classes significantly differed in all symptom domains (*p*_FDR_s < .001–.017, *d*’s = 0.27–0.50) with the exception of oppositional behaviour (*p* = .07). Similarly, class 4 also differed significantly from class 2 on almost all measures of ASD, ADHD, and comorbid symptoms (*p*_FDR_s < .003–.024, *d*’s = 0.23–0.30) except for oppositional symptoms (*p* = .07) and fear of changes (*p* = .08). Class 1 and 2 differed in several ASD symptoms. Class 3 did not differ significantly from any of the classes, potentially due to the small sample size.

The pattern of results did not change, when age, sex, and IQ were accounted for, but the significance of the majority of these class differences was attenuated (Additional file 1: Table S7). Class 1–class 4 differences in two CSBQ items remained significant (not tuned in: *p*_FDR_ = .002, *d* = 0.34; Tendency to withdraw: *p*_FDR_ = .002, *d* = 0.35).

Diagnostic profiles of members of the classes are given in Fig. 1, which provides weighted percentages to account for differences in the numbers of participants in the diagnostic groups. Pure and comorbid ASD and ADHD patients, their unaffected siblings, and controls all were present in every class. The average to strong performing classes (classes 1 and 2) consisted of 28.3% and 15.3% controls, respectively, class 3 had 10.9% controls, but also the weakest performing class (class 4) contained 10.1% of controls. With regard to patients, class 4 consisted of 25.1% ASD + ADHD patients, 17.2% ASD-only patients, and 23.8% ADHD-only patients. Class 1 contained 11.4% ASD + ADHD patients, 9.0% ASD-only patients, and 15.2% ADHD-only patients. Similarly, class 2 also had substantial proportions of patients, with 16.5% ASD + ADHD patients, 19.3% ASD-only patients, and 13.2% ADHD-only patients. Nonetheless, within-class proportions comparison showed that only class 1 had significantly more controls than ASD + ADHD patients (*X*^2^(1) = 7.41, *p*_FDR_ = .045, *d* = 0.68) and ASD-only patients (*X*^2^(1) = 9.76, *p*_FDR_ = .03, *d* = 0.76) after FDR correction (Additional file 1: Table S8).

### How do patients with and without emotion recognition impairments differ?

To examine how patients with and without emotion recognition impairments diverged in their symptoms, post hoc analyses comparing the patients from class 4 with those from the best performing classes 1 and 2 were performed. Again, in these analyses, the phenotypic profiles of classes were compared using mixed models and corrected for age and familial effects. ASD + ADHD, ASD-only, and ADHD-only patients in classes 4, 1, and 2 did not significantly differ in ASD- or ADHD-related symptom scores (see Additional file 1: Figure S2). These results did not change when age, sex, and IQ were accounted for.

## Discussion

In this study, we aimed to investigate emotion recognition as a transdiagnostic phenotype in order to understand the heterogeneity and comorbidity of ADHD and ASD. We did so by identifying emotion recognition subtypes in pure and comorbid ASD and ADHD patients, their unaffected siblings, and healthy controls. This was done using FMM, which is powerful in integrating variable-based data reduction (factor analysis) with a person-based identification of qualitatively different subtypes (latent class analysis). We explored whether the identified subtypes provide insight into the heterogeneity and comorbidity of ASD and ADHD. Analyses revealed four groups of participants: an average to strong performing class with reasonably fast and accurate performance across visual and auditory domains (class 2), a class with impulsive auditory recognition yet reasonably fast and accurate in their visual emotion recognition (class 1), a poor performing class with slow and inaccurate performance across both domains (class 4), and an intermediate class with a large speed-accuracy trade-off in the visual domain (class 3). None of the identified classes or related emotion recognition impairments in either modality were particularly linked to ASD or ADHD. All classes contained patients from all three diagnostic categories (ASD-only, ADHD-only, and ASD + ADHD) as well as their unaffected siblings and controls. Emotion recognition impairments were similarly frequent in ASD as in ADHD and ASD + ADHD, with 17.2% of ASD patients, 23.8% of ADHD patients, and 25.1% of patients with comorbid ASD and ADHD falling into the weakest performing class. At the level of quantitative measures of disease-related behaviour, the relationship between the two ADHD symptom domains inattention vs hyperactivity/impulsivity and class status was comparable, indicating emotion recognition problems linked similarly to both ADHD symptom domains. There was a clear enrichment of ASD- and ADHD-related symptoms seen in the worst-performing class 4. However, post hoc tests demonstrated that although these symptoms are associated with emotion recognition impairments, there is certainly no one-to-one relationship.

The poorest performing emotion recognition class (class 4) differed from the two good/average performing classes (classes 1 and 2) in quantitative measures, with higher levels of ASD, ADHD, and comorbid symptoms being present in class 4. Previous studies [12, 26] suggested that behavioural symptoms, such as inattention, are important contributors to emotion recognition dysfunction. Our results partially support this notion, given that higher levels of ASD, ADHD, and comorbid symptoms were present in the weakest performing class. Yet, the pure and comorbid ASD and ADHD patients in the strong to average performing classes (classes 1 and 2) did not differ significantly in quantitative measures of ASD and ADHD symptoms compared to the weakest class (class 4), indicating that the relationship between ASD and ADHD behavioural symptoms and emotion recognition dysfunction is not 1:1. Furthermore, our findings speak to the presence of cognitive heterogeneity in both ASD and ADHD, which corroborates previous findings of multiple developmental pathways in ADHD [4, 50] and ASD [5, 69]. Moreover, none of the identified emotion recognition subtypes were specific to ASD or ADHD, neither in symptoms nor in diagnosis. This lack of specificity, combined with observed associations with comorbid symptoms, suggests that emotion recognition dysfunction is a trait that can be utilised to understand the co-occurrence of other disorders that are frequently comorbid with ASD and ADHD (e.g. bipolar disorder, anxiety disorders, conduct disorder) and that are also marked by emotion recognition dysfunction [6].

Despite the commonalities in findings for emotion recognition problems in relation to both quantitative ASD and ADHD symptom measures as well as diagnostic/categorical measures, it is possible that the mechanisms underlying poor emotion recognition in ASD and ADHD differ. For example, individuals with ASD allocated to the weakest emotion recognition class may have a primary emotion recognition deficit, whereas individuals with ADHD allocated to the same class might have more general information processing difficulties, with emotion recognition problems being secondary to that. To address this issue, mechanistic measures are needed, foremost functional brain activation whilst performing emotion recognition tasks. Unfortunately, in the current study, these data were not available. However, even if underlying mechanisms contributing to a poor emotion recognition performance may be partly dissimilar in individuals with ASD and ADHD, the end result is that on a performance level, a large proportion of individuals with ADHD performs on the worst level as measured by the tasks administered in this study. This likely translates to social difficulties in daily life, where a poor registration of emotional expressions of other people (whether due to primary emotion recognition difficulties or secondary general information processing difficulties) is likely to interfere with social interaction. In conclusion, although functional brain imaging measures are needed to shed light on the issue of (non-) overlapping mechanisms underlying poor emotion recognition in individuals with ASD and ADHD, on a performance level, much similarities are observed with poor emotion recognition abilities present in a substantial proportion of individuals with ASD and ADHD alike.

This study demonstrates the benefits of utilising a model-based approach to gain insight into the comorbidity of disorders, in this case, ASD and ADHD. Our model suggests that emotion recognition dysfunction may not be a feature that distinguishes between ASD and ADHD. Previous literature has shown emotion recognition to be a plausible endophenotype [12, 48] and that the development of social cognition is suggested to be functionally dependent on the maturation of cognitive skills, and possibly vice versa [47, 70, 71]. Therefore, our findings, combined with current literature, could indicate support for models like the step-endophenotype framework [5]. This model poses that below a certain threshold, an individual’s risk is low; yet, once a threshold has been reached, the risk markedly increases. In this case, it may be hypothesised that—similar to cognitive dysfunction—emotion recognition dysfunction only increases the likelihood of neurodevelopmental symptoms and disorders in combination with other risk factors and/or after a certain threshold has been exceeded. However, much remains to be investigated regarding this relationship between emotion recognition, these neurodevelopmental disorders and their symptoms, as well as other risk factors. The identified subtypes may differ in neural correlates, genetics, and their developmental trajectories, which may affect their response to treatment. Studies including longitudinal designs are required to further clarify the role of emotion recognition in its effects on developmental psychopathology.

This study has several strengths, including the use of both categorical and dimensional measures of psychopathology and analyses, employing validated emotion recognition paradigms, and the inclusion of a large sample size containing patients, unaffected siblings, and controls. This has enabled comparisons of both quantitative and qualitative differences in emotion recognition impairments across ASD and ADHD and the assessment of the heterogeneity of such impairments across the entire breadth of the symptom distribution. However, even with our large sample size, the least prevalent class (class 3) reached only a limited size. For this class, power was limited to investigate links to diagnostic status and quantitative behavioural measures. This study was also limited by the type of emotion recognition tasks used. Although these tasks have been validated [65], they assess visual and auditory emotion recognition separately. Future studies of simultaneous visual and auditory emotion recognition could provide more insight into multimodal emotion processing abilities. Another potential limitation of our study was that all of our participants had an IQ higher than 70, limiting the generalisability of the results to individuals with an IQ below 70. IQ also significantly differed between subtypes, with the average-strong performing classes (classes 1 and 2) having higher IQs. Furthermore, IQ was associated with the accuracy of emotion recognition, corroborating with reports that IQ and emotion recognition abilities are intertwined [72, 73]. However, controlling for IQ differences did not alter the findings, indicating that class differences were not driven by overall cognitive performance differences. The male to female ratio was more balanced in the group of individuals with ADHD than it was in the group of individuals with ASD. However, since results were analysed with gender-corrected means, it is unlikely that this has influenced the outcome of the clustering analysis. Although we used FMM because of its many advantages, there are other methodologies that can investigate the heterogeneity of these disorders. For example, Lombardo et al. [74] used hierarchical clustering of participants patterns of response on the Reading the Minds Eye Test (RMET) to understand the heterogeneity of metalizing in ASD and typically developing participants. This study found response patterns specific to ASD subgroups and other response patterns specific to typically developing participants. Though this was not the aim of the current study, elucidating diagnostic specific response patterns are potentially informative of diagnostic differentiating factors. As such, the advantages and disadvantages of each modelling technique should be considered in relation to the aims of a study.

The findings of this study have clear clinical implications. Emotion recognition dysfunction cannot be used either to confirm or disconfirm the presence of ASD and/or ADHD. However, this does not mean that emotion recognition impairments should not be assessed and treated when necessary in both ASD and ADHD. As demonstrated, emotion recognition impairments are at least as important in ADHD as they are in ASD, particularly as these impairments show links to cognitive functioning and are likely to contribute to emotion dysregulation, both of which can be identified in ASD and ADHD. Therefore, for some individuals with ASD and/or ADHD, the inclusion of emotion recognition skills training could be highly beneficial.

## Conclusions

This study identified emotion recognition subtypes in patients with pure and comorbid ASD and ADHD, their unaffected siblings, and healthy controls using FMM. Emotion recognition dysfunction behaves as a risk factor for developing ASD and/or ADHD, although heterogeneity of impairments across clinical groups and controls clearly shows that there is no 1:1 relationship with ASD and/or ADHD symptoms. The observed classes with differential emotion recognition profiles warrant further investigation, as they could differ in neural correlates and genetics, and prognosis may be different. We conclude that emotion recognition dysfunction should be considered when assessing and treating both ASD and ADHD, demonstrating the need to broadly assess an individual’s strengths and weaknesses to provide optimal care.

## Additional file

Additional file 1: Emotion Recognition Subtyping Supplement. (DOCX 954 kb)

## Abbreviations

ADHD: Attention-deficit/hyperactivity disorder
ADI—R: Autism Diagnostic Interview—Revised
AIC: Akaike information criteria
ANT: Amsterdam Neuropsychological Task
AP: Affective Prosody task
ASD + ADHD: Comorbid autism spectrum disorders and attention-deficit/hyperactivity disorder
ASD: Autism spectrum disorders
BIC: Bayesian information criteria
BOA: Biological Origins of Autism study
CFA: Confirmatory factor analysis
CPRS: Conners Parents Rating Scale—Revised
CTRS: Conners Teachers Rating Scale—Revised
FDR: False discovery rate
FMM: Factor mixture modelling
FSIQ: Full Scale Intelligence Quotient
IFE: Identification of Emotional Expressions task
IMAGE: International Multicenter ADHD Genetics study
IQ: Intelligence quotient
K-SADS: Schedule for Affective Disorders and Schizophrenia for School-Age Children (Present and Lifetime version)
LCA: Latent class analysis
PACS: Parental Account of Childhood Symptoms
*p*FDR: FDR-corrected *p* value (*q* value)
RMET: Reading the Minds Eye Test
SCQ: Social Communication Questionnaire
WAIS-III: Wechsler Adult Intelligence Scale III
WISC-III: Wechsler Intelligence Scale for Children version III
