# Supplementary material for: An emotion recognition subtyping approach to studying the heterogeneity and comorbidity of autism spectrum disorders and attention-deficit/hyperactivity disorder
Source: J Neurodev Disord. 2018 Nov 15;10:31. doi: 10.1186/s11689-018-9249-6 (PMC6238263; doi:10.1186/s11689-018-9249-6)
Supplement: Supplementary file 1 — Emotion Recognition Subtyping Supplement. (DOCX 954 kb) [file 11689_2018_9249_MOESM1_ESM.docx]

**An emotion recognition subtyping approach to studying the heterogeneity and comorbidity of Autism Spectrum Disorders and Attention-Deficit/Hyperactivity Disorder- Additional file 1**

**Method**

Diagnostic Assessment – additional information

*ADHD screening and diagnosis*

All participants were screened for ADHD using the long version of the Conners Rating Scale - Revised (CRS-R:L;) [1]. Questionnaires were completed by parents (CPRS-R:L) and teachers (CTRS-R:L) and filled out about a period without medication. The following scales were used: DSM Inattentive behaviour (scale L), DSM Hyperactive/Impulsive behaviour (scale M), and DSM Total (scale N).

To determine ADHD diagnoses in both cohorts, a diagnostic algorithm was used to combine symptom counts on a semi-structured diagnostic interview and Conners’ ADHD questionnaires, both providing operational definitions of each of the 18 behavioural symptoms defined by the DSM-IV (American Psychiatric Association, 2014). All participants were administered the Dutch translation of the Schedule for Affective Disorders and Schizophrenia for School-Age Children - Present and Lifetime Version (K-SADS; in NeuroIMAGE) [2] or the PACS (in BOA), carried out by trained professionals. Both the parents and the child, if ≥12 years old, were interviewed separately and were initially only administered the ADHD screening interview. Participants with elevated scores on any of the screen items were administered the full ADHD section. Symptoms of the CTRS-R:L were only used in the algorithm if at least two symptoms were reported on this questionnaire. Participants with a combined symptom count of ≥6 symptoms of hyperactive/impulsive behaviour and/or inattentive behaviour were diagnosed with ADHD, provided they: a) met the DSM-IV criteria for pervasiveness and impact of the disorder (measures derived from the K-SADS/PACS), b) showed an age of onset before 12 years (following the proposed changes for the DSM-V; see [3]), derived from the K-SADS/PACS, and c) the child received a *T* ≥63 on at least one of the three DSM ADHD scales on either one of the Conners ADHD questionnaires. Inconsistent cases were evaluated by a team of trained experts (consisting of psychiatrist JB and 8 psychologists), in order to derive a consensus diagnosis.

Unaffected participants were required to receive a *T*-score <63 on each of the scales of both the Conners ADHD questionnaires (NeuroIMAGE and BOA), and have ≤3 symptoms derived from the combined symptom counts of the K-SADS and CTRS-R:L (NeuroIMAGE) or did not fulfil the PACS criteria (BOA).

*ASD screening and diagnosis*

All participants with a clinical ASD diagnosis and their siblings (BOA) were similarly screened for ASD using the Social Communication Questionnaire (SCQ) [4]. The questionnaire was completed by parents and teachers and filled out about a period without medication. A cut-off score of ≥11 was considered as clinical. For all participants scoring in the clinical range, a formal diagnosis of ASD was made by a certified clinician using the Autism Diagnostic Interview - Revised (ADI-R [5]; procedure fully described [6]). Children with a clinical diagnosis, who did not fulfil ADI-R criteria, were excluded along with their parents and siblings. All siblings either scoring below or above cut-off on the SCQ, who did not fulfil ADI-R criteria, were labelled as unaffected siblings. Participants from NeuroIMAGE, who were screened as positive on the SCQ (SCQ score >15), were administered the ASD section of the PACS, and excluded from participation. Healthy control children were similarly screened for ASD and ADHD and were required to score below cut-off on the SCQ (<11) and both Conners’ ADHD questionnaires (<63) in order to be involved in this study.

*Quantitative symptom severity*

Quantitative ASD symptom severity was determined in both cohorts using the Children’s Social Behaviour Questionnaire (CSBQ). The CSBQ is a parental 49-item questionnaire measuring social problem behaviour with adequate reliability and validity [7]. The CSBQ ‘ASD Composite score’ (aggregated score of the four subscales: reduced contact and social interests, difficulties in understanding social information, stereotyped behaviour, and fear of and resistance to changes) was used in [8]. The CRS R:L were used to assess ADHD, anxiety (scale D) and oppositional behaviour (scale A) symptoms.

*Emotion recognition task*

**Figure S1**. Photos of happy, sad, angry and fearful expressions shown in the Identification of Facial Emotions (IFE) task from the Amsterdam Neuropsychological Tasks battery [9].


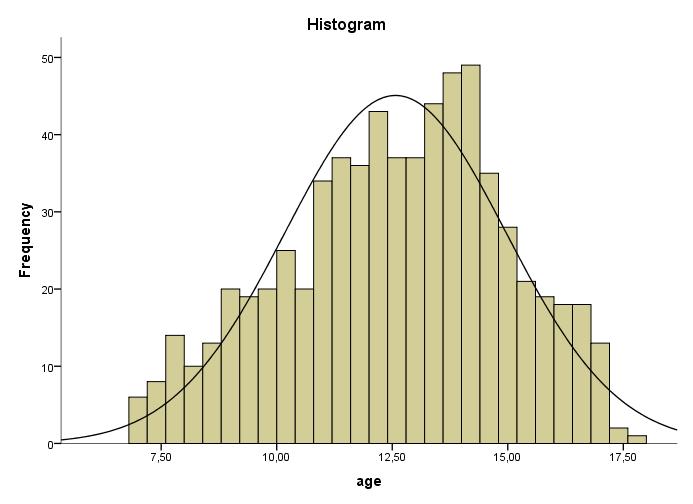


**Figure S2.** Distribution of age of participants from the BOA and NeuroIMAGE cohorts included in the current study.

Model Fit Criteria

There are several model fit indices available including, Akaike Information Criteria (AIC), Bayesian Information Criteria (BIC), adjusted Bayesian Information Criteria (aBIC), Entropy, and Lo-Mendell Rubin Adjusted LRT test. These fit criteria have been reported to have their advantages and disadvantages [10,11,12,13,14] and therefore, these criteria were used in combination when selecting a model. The four factors that were previously identified represent (1) speed of identification of facial emotional expressions, (2) accuracy of identification of facial emotional expressions, (3) speed of prosody, and (4) accuracy of prosody. The Latent Class Analysis (LCA) of both cohorts combined was performed (Table S2), and these results were then used to guide the fitting of the FMM (Table S3).

**Results**

**Model Fit**

**Table S1.** Fit of Confirmatory Factor Analysis (CFA) model

| Factors | AIC | BIC | Satorra-Bentler Chi-square *p* value | RMSEA | CFI | TLI |
| --- | --- | --- | --- | --- | --- | --- |
| 1 | 25630.828 | 25892.682 | <.001 | 0.137 | 0.497 | 0.491 |
| 2 | No Convergence | | | | | |
| 3 | 24612.809 | 24919.809 | <.001 | 0.056 | 0.920 | 0.914 |
| 4 | 24493.911 | 24837.029 | <.001 | 0.033 | 0.974 | 0.971 |

*N.B.* AIC-Akaike Information Criteria, BIC-Bayesian Information Criteria, RMSEA-Root Mean Square error of Approximation, CFI-Comparative Fit Index, TLI-Tucker Lewis Index.

**Table S2.** Latent Class Analysis model

| Classes | AIC | BIC | aBIC | Entropy | Lo-Mendell Rubin Adjusted LRT test *p*-value | | |
| --- | --- | --- | --- | --- | --- | --- | --- |
| 3 | 25533.90 | 25795.76 | 25611.60 | 0.79 | | 0.038 |  |
| 4 | 25323.63 | 25653.21 | 25421.42 | 0.80 | | 0.036 | |
| 5 | 25191.64 | 25588.93 | 25309.52 | 0.80 | | 0.378 | |

*N.B.* AIC-Akaike Information Criteria, BIC-Bayesian Information Criteria, aBIC-Adjusted Bayesian Information Criteria.

| Factors | Classes | AIC | BIC | aBIC | Entropy |
| --- | --- | --- | --- | --- | --- |
| 4 | 3 | 25533.90 | 25588.93 | 25611.60 | 0.79 |
| 4 | 4 | 25323.63 | 25653.21 | 25421.42 | 0.80 |
| 4 | 5 | 25191.64 | 25588.93 | 25309.52 | 0.80 |

**Table S3.** Factor Mixture Model

*N.B.* AIC-Akaike Information Criteria, BIC-Bayesian Information Criteria, aBIC-Adjusted Bayesian Information Criteria.

**Table S4.** Characteristics of each diagnostic group.

| (N / % of class) | Age (M/*SD*) | IQ (M/*SD*) | Males | Cohort |
| --- | --- | --- | --- | --- |
| ASD+ADHD | **12.14** /*2.79* | **102.36 /** 13.15 | 56 / 88% | 100% BOA |
| ASD-only | **12.32** /*2.48* | **101.51 /** 14.67 | 69 / 78% | 100% BOA |
| ADHD-only | **12.67** / *1.71* | **99.49** / *14.13* | 57 / 51% | 100% NeuroIMAGE |
| ASD(+ADHD) unaffected siblings | **11.77** / *2.79* | **105.73 /** 12.63 | 53 / 43% | 100% BOA |
| ADHD-only unaffected siblings | **12.81** /*1.63* | **99.61** / *14.42* | 26 / 38% | 100% NeuroIMAGE |
| Controls | **13.11** / *2.35* | **105.15 /** 12.42 | 110 / 50% | 57.7% BOA  42.3% NeuroIMAGE |

**Table S5.** Characteristics of each emotion recognition class

| (N / % of class) | Class 1 N=173  Average visual, impulsive auditory emotion recognition | Class 2 N=303  Average-strong visual & auditory emotion recognition | Class 3 N=50  Impulsive & imprecise visual, average auditory emotion recognition | Class 4 N=149  Weak visual & auditory emotion recognition |
| --- | --- | --- | --- | --- |
| ASD+ADHD | 10 / 5.78% | 29 / 9.57% | 2 / 4% | 23 / 15.44% |
| ASD-only | 11 / 6.36% | 47 / 15.51% | 9 / 18% | 22 / 14.77% |
| ADHD-only | 23 / 13.30% | 40 / 13.20% | 10 / 20% | 38 / 25.50% |
| ASD(+ADHD) unaffected siblings | 23 / 13.30% | 63 / 20.79% | 12 / 24% | 24 / 16.11% |
| ADHD-only unaffected siblings | 21 / 12.14% | 32 / 10.56% | 6 / 12% | 10 / 6.71% |
| Controls | 85 / 49.13% | 92 / 30.36% | 11 / 22% | 32 / 21.48% |
| Males | 76 / 44% | 167 / 55% | 31 / 62% | 97 / 65% |
| Age (M/*SD*) | **12.65 /** *2.48* | **12.69 /** *2.29* | **11.88 /** *2.57* | **12.46 /** *2.41* |
| IQ (M/*SD*) | **105.04 /** *14.39* | **104.27 /** *14.79* | **101.65 /** *13.65* | **95.09 /** *12.93* |

**Table S6.** FDR-corrected *P*-values of the pairwise comparison of emotion recognition factors across classes.

|  | Class 1: Average visual, impulsive auditory emotion recognition  Comparison with: | | | Class 2: Average-strong visual & auditory emotion recognition  Comparison with: | | Class 3: Impulsive & imprecise visual, average auditory emotion recognition  Comparison with: |
| --- | --- | --- | --- | --- | --- | --- |
|  | Class 2: average-strong visual & auditory emotion recognition | Class 3: impulsive & imprecise visual, average auditory emotion recognition | Class 4: weak visual & auditory emotion recognition | Class 3: impulsive & imprecise visual, average auditory emotion recognition | Class 4: weak visual & auditory emotion recognition | Class 4: weak visual & auditory emotion recognition |
| Speed Visual Recognition | <.001 | <.001 | <.001 | <.001 | <.001 | <.001 |
| Accuracy Visual Recognition | <.001 | <.001 | <.001 | <.001 | <.001 | <.001 |
| Speed Auditory Recognition | <.001 | <.001 | <.001 | .87 | <.001 | <.001 |
| Accuracy Auditory Recognition | <.001 | <.001 | .002 | <.001 | <.001 | <.001 |

*N.B.* Age covariate included.

**Table S7.** FDR-corrected *P*-values of the pairwise comparison of emotion recognition factors across classes.

|  | Class 1: Average visual, impulsive auditory emotion recognition  Comparison with: | | | Class 2: Average-strong visual & auditory emotion recognition  Comparison with: | | Class 3: Impulsive & imprecise visual, average auditory emotion recognition  Comparison with: |
| --- | --- | --- | --- | --- | --- | --- |
|  | Class 2: average-strong visual & auditory emotion recognition | Class 3: impulsive & imprecise visual, average auditory emotion recognition | Class 4: weak visual & auditory emotion recognition | Class 3: impulsive & imprecise visual, average auditory emotion recognition | Class 4: weak visual & auditory emotion recognition | Class 4: weak visual & auditory emotion recognition |
| Speed Visual Recognition | <.001 | <.001 | <.001 | <.001 | <.001 | <.001 |
| Accuracy Visual Recognition | <.001 | <.001 | <.001 | <.001 | <.001 | <.001 |
| Speed Auditory Recognition | <.001 | <.001 | <.001 | .92 | <.001 | .001 |
| Accuracy Auditory Recognition | <.001 | <.001 | <.001 | <.001 | <.001 | <.001 |

*N.B.* Age, Sex and IQ covariates included.

**Table S8.** Speed-accuracy trade-offs in the classes.

|  | | Class 1: Average visual, impulsive auditory emotion recognition  Comparison with: | Class 2: Average-strong visual & auditory emotion recognition  Comparison with: | Class 3: Impulsive & imprecise visual, average auditory emotion recognition  Comparison with: | | Class 4: Weak visual & auditory emotion recognition  Comparison with: | | |
| --- | --- | --- | --- | --- | --- | --- | --- | --- |
| Visual Speed vs Visual Accuracy | .001 | | <.001 | | <.001 | | .67 |  |
| Auditory Speed vs Auditory Accuracy | <.001 | | <.001 | | .003 | | .029 |  |
| Visual Speed vs Auditory Speed | <.001 | | .001 | | <.001 | | <.001 |  |
| Visual Accuracy vs Auditory Accuracy | <.001 | | .001 | | <.001 | | .004 |  |

**Table S9.** FDR-corrected *p*-values of the pairwise comparisons of Age, IQ, proportion of males, ASD, ADHD, and related symptoms across classes

|  | | | Class 1: Average visual, impulsive auditory emotion recognition  Comparison with: | | | | Class 2: Average-strong visual & auditory emotion recognition  Comparison with: | | Class 3: Impulsive & imprecise visual, average auditory emotion recognition  Comparison with: |
| --- | --- | --- | --- | --- | --- | --- | --- | --- | --- |
|  | | | Class 2: Average-strong visual & auditory emotion recognition | Class 3: Impulsive & imprecise visual, average auditory emotion recognition | Class 4: Weak visual & auditory emotion recognition | Class 3: Impulsive & imprecise visual, average auditory emotion recognition | Class 4: Weak visual & auditory emotion recognition | Class 4: Weak visual & auditory emotion recognition |  |
| IQ | | | .62 | .45 | <.001 | .52 | <.001 | .006 |  |
| Age | | | .86 | .14 | .55 | .14 | .48 | .29 |  |
| % Male | | | .54 | .24 | .24 | .62 | .54 | .79 |  |
|  | | |  |  |  |  |  |  |  |
| Conners Reports (age covariate included)  *(age, sex, and IQ covariates included)* | | |  |  |  |  |  |  |  |
| Inattention | Parent | | .30  *(.32)* | .18  *(.20)* | **.006**  *(.03)* | .37  *(.40)* | **.024**  *(.12)* | .53  *(.74)* |  |
|  | Teacher | | .38  *(.42)* | .25  *(.31)* | **.002**  *(.04)* | .38  *(.42)* | **.006**  *(.09)* | .38  *(.65)* |  |
| Hyperactivity/impulsivity | Parent | | .60  *(.83)* | .60  *(.83)* | **.002**  *(.04)* | .83  *(.94)* | **.003**  *(.04)* | .16  *(.31)* |  |
|  | Teacher | | 1  *(.81)* | .67  *(.72)* | .20  *(.18)* | .67  *(.72)* | .20  *(.18)* | .67  *(.75)* |  |
| Oppositional | Parent | | .60  *(.67)* | .07  *(.12)* | .07  *(.12)* | .08  *(.12)* | .07  *(.13)* | .60  *(.58)* |  |
|  | Teacher | | .71  *(.90)* | **.027**  *(.01)* | .65  *(.56)* | **.018**  *(.01)* | .45  *(.52)* | .06  *(.04)* |  |
| Anxiety | Parent | | .052  *(.18)* | .10  *(.22)* | **<.001**  *(.02)* | .54  *(.69)* | **.018**  *(.22)* | .35  *(.69)* |  |
|  | Teacher | | .46  *(.46)* | .41  *(.46)* | .41  *(.46)* | .41  *(.46)* | .46  *(.78)* | .46  *(.46)* |  |
| Total | Parent | | .37  *(.46)* | .31  *(.46)* | **.001**  *(.024)* | .47  *(.53)* | **.003**  *(.04)* | .31  *(.46)* |  |
|  | Teacher | | .41  *(.50)* | .39  *(.42)* | **.006**  *(.06)* | .41  *(.50)* | **.012**  *(.10)* | .41  *(.60)* |  |
| CSBQ (age covariate included)  *(age, sex and IQ covariates included)* |  | |  |  |  |  |  |  |  |
| Not tuned in | | | **.016**  *(.05)* | .053  *(.11)* | **<.001**  ***(.002)**** | .053  *(.64)* | **.015**  *(.11)* | .34  *(.60)* |  |
| Tendency to withdraw | | | **.012**  ***(.036)**** | .11  *(.30)* | **.017**  ***(.002)**** | .98  *(.76)* | **.018**  *(.15)* | .14  *(.27)* |  |
| Orientation problems | | | **.015**  *(.08)* | .21  *(.56)* | **<.001**  *(.04)* | .80  *(.56)* | **.018**  *(.41)* | .11  *(.41)* |  |
| Not understanding | | | **.028**  *(.11)* | .32  *(.55)* | **<.001**  *(.02)* | .76  *(.55)* | **.012**  *(.24)* | .07  *(.24)* |  |
| Stereotypic behaviour | | | .09  *(.28)* | .20  *(.51)* | **<.001**  *(.04)* | .81  *(.96)* | **.012**  *(.28)* | .20  *(.43)* |  |
| Fear of change | | | .08  *(.20)* | .27  *(.48)* | **.002**  *(.07)* | .98  *(.83)* | .08  *(.48)* | .27  *(.48)* |  |
| Total ASD core symptoms | | | **.02**  *(.12)* | .07  *(.25)* | **<.001**  ***(.006)**** | .59  *(.81)* | **.006**  *(.16)* | .23  *(.49)* |  |

*N.B.* * remained significant after including age, sex, and IQ as covariates.

**Table S10.** FDR-corrected *p*-values for within-class weighted proportions

| Class 1 |  | FDR-corrected *p*-value |
| --- | --- | --- |
| ASD+ADHD | ASD-only | .70 |
|  | ADHD-only | .54 |
|  | ASD(+ADHD) unaffected siblings | .63 |
|  | ADHD unaffected siblings | .14 |
|  | Controls | .045 |
| ASD-only | ADHD-only | .41 |
|  | ASD(+ADHD) unaffected siblings | .45 |
|  | ADHD unaffected siblings | .10 |
|  | Controls | .03 |
| ADHD-only | ASD(+ADHD) unaffected siblings | .85 |
|  | ADHD unaffected siblings | .42 |
|  | Controls | .14 |
| ASD(+ADHD) unaffected siblings | ADHD unaffected siblings | .39 |
|  | Controls | .12 |
| ADHD unaffected siblings | Controls | .54 |
|  |  |  |
| Class 2 |  | **FDR-corrected *p*-value** |
| ASD+ADHD | ASD-only | 0.85 |
|  | ADHD-only | 0.85 |
|  | ASD(+ADHD) unaffected siblings |  |
|  | ADHD unaffected siblings | 1 |
|  | Controls | 0.85 |
| ASD-only | ADHD-only | 0.85 |
|  | ASD(+ADHD) unaffected siblings | 1 |
|  | ADHD unaffected siblings | 0.85 |
|  | Controls | 0.85 |
| ADHD-only | ASD(+ADHD) unaffected siblings | 0.85 |
|  | ADHD unaffected siblings | 0.85 |
|  | Controls | 0.85 |
| ASD(+ADHD) unaffected siblings | ADHD unaffected siblings | 0.85 |
|  | Controls | 0.85 |
| ADHD unaffected siblings | Controls | 0.85 |
|  |  |  |
| Class 3 |  | **FDR-corrected *p*-value** |
| ASD+ADHD | ASD-only | 0.06 |
|  | ADHD-only | 0.06 |
|  | ASD(+ADHD) unaffected siblings | 0.06 |
|  | ADHD unaffected siblings | 0.07 |
|  | Controls | 0.58 |
| ASD-only | ADHD-only | 0.88 |
|  | ASD(+ADHD) unaffected siblings | 0.88 |
|  | ADHD unaffected siblings | 0.88 |
|  | Controls | 0.17 |
| ADHD-only | ASD(+ADHD) unaffected siblings | 0.88 |
|  | ADHD unaffected siblings | 0.88 |
|  | Controls | 0.23 |
| ASD(+ADHD) unaffected siblings | ADHD unaffected siblings | 0.88 |
|  | Controls | 0.19 |
| ADHD unaffected siblings | Controls | 0.27 |
|  |  |  |
| Class 4 |  | **FDR-corrected *p*-value** |
| ASD+ADHD | ASD-only | 0.36 |
|  | ADHD-only | 0.95 |
|  | ASD(+ADHD) unaffected siblings | 0.23 |
|  | ADHD unaffected siblings | 0.06 |
|  | Controls | 0.06 |
| ASD-only | ADHD-only | 0.41 |
|  | ASD(+ADHD) unaffected siblings | 0.68 |
|  | ADHD unaffected siblings | 0.33 |
|  | Controls | 0.33 |
| ADHD-only | ASD(+ADHD) unaffected siblings | 0.26 |
|  | ADHD unaffected siblings | 0.06 |
|  | Controls | 0.06 |
| ASD(+ADHD) unaffected siblings | ADHD unaffected siblings | 0.52 |
|  | Controls | 0.52 |
| ADHD unaffected siblings | Controls | 1 |

**Figure S3.** Post-hoc comparisons of symptoms seen in probands in class 4 (ASD+ADHD N=10; ASD-only N=11; ADHD-only N=23) and class 1 (ASD+ADHD N=29; ASD-only N=46; ADHD-only N=39; panel A) and class 2 (ASD+ADHD N=23; ASD-only N=22; ADHD-only N=37; panel B). Bars represent mean z-score for total symptoms (+/-1 S.E.).


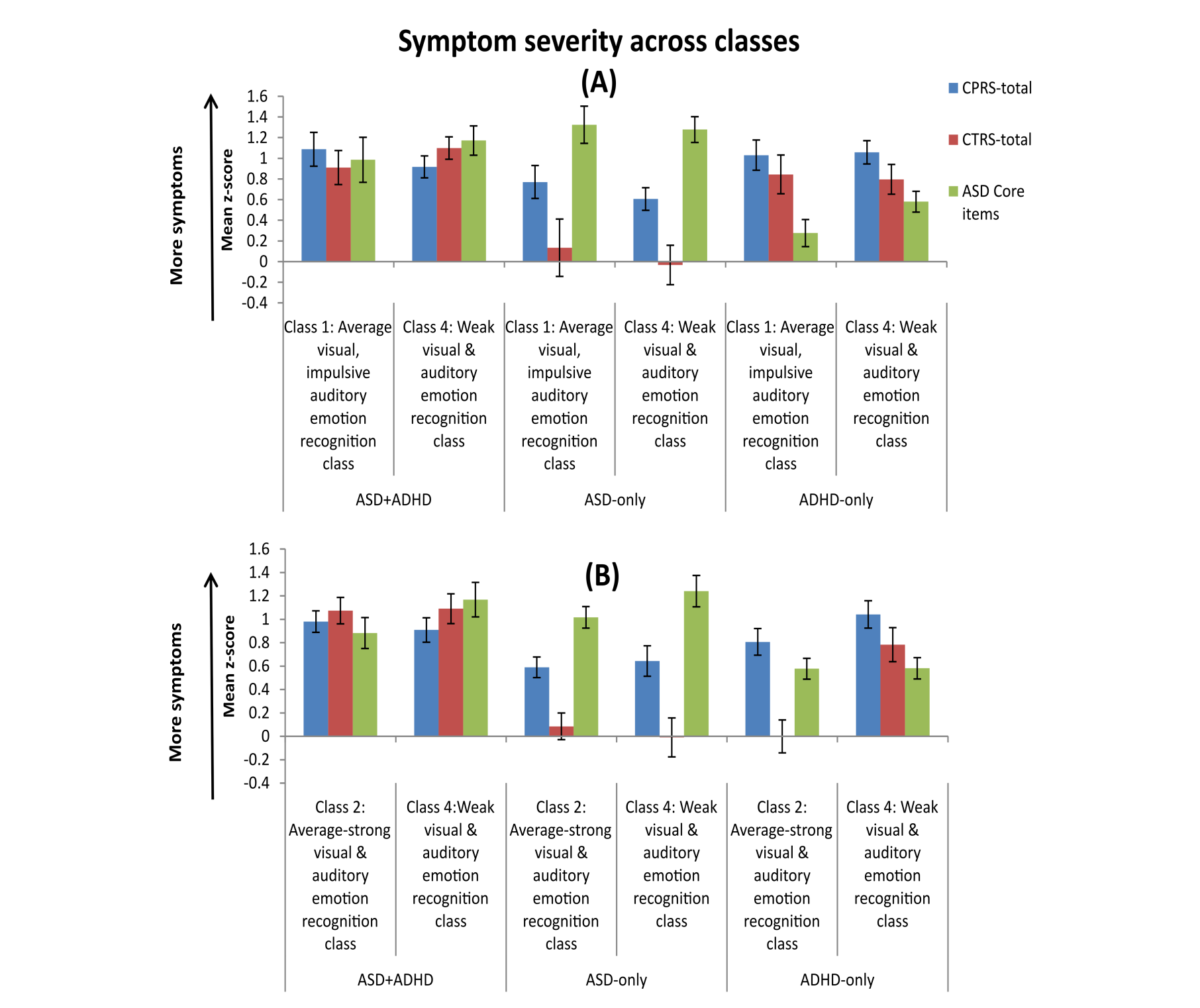


**References**

1. Conners, C. K. *Conners' Parent Rating Scale--Revised (L)*. 1997.
2. Kaufman, J., Birmaher, B., Brent, D., Rao, U., Flynn, C., Moreci, P., Williamson, D. & Ryan, N. Schedule for Affective Disorders and Schizophrenia for School-Age Children Present and Lifetime version (K-SADS-PL): Initial reliability and validity data. *J Am Acad Child Adolesc Psychiatry.* 1997;36:980-988.
3. Polanczyk, G., Caspi, A., Houts, R., Kollins, S. H., Rohde, L. A. & Moffitt, T. E. Implications of Extending the ADHD Age-of-Onset Criterion to Age 12: Results from a Prospectively Studied Birth Cohort. *J Am Acad Child Adolesc Psychiatry* .2010;49:210-216.
4. Rutter, M. The social communication questionnaire: Manual. 2003
5. Le Couteur, A., Lord, C. & Rutter, M. The autism diagnostic interview-revised (ADI-R). *Los Angeles, CA: Western Psychological Services*. 2003.
6. van Steijn, D. J., Richards, J. S., Oerlemans, A. M., de Ruiter, S. W., van Aken, M. A. G., Franke, B., Buitelaar, J. K. & Rommelse, N. N. J. The co-occurrence of autism spectrum disorder and attention-deficit/hyperactivity disorder symptoms in parents of children with ASD or ASD with ADHD. *J Child Psychol Psychiatry .*2012;53;954-963.
7. Hartman, C. A., Luteijn, E., Serra, M. & Minderaa, R. Refinement of the children's social behavior questionnaire (CSBQ): An instrument that describes the diverse problems seen in milder forms of PDD. *J*Autism*Dev Disord.* 2006;36: 325-342.
8. . t Hart-Kerkhoffs, L. A., Jansen, L. M., Doreleijers, T. A., Vermeiren, R., Minderaa, R. B. & Hartman, C. A. Autism Spectrum Disorder Symptoms in Juvenile Suspects of Sex Offenses. *J Clin Psychiatry* 2009;70:266-272.
9. De Sonneville, L. Amsterdam Neuropsychological Tasks: A computer-aided assessment program. *Comp Psych.* 1999;6:87-203.
10. Lo, Y. T., Mendell, N. R. & Rubin, D. B. Testing the number of components in a normal mixture. *Biometrika* 2001;88:767-778.
11. Lubke, G. & Muthen, B. O. Performance of factor mixture models as a function of model size, covariate effects, and class-specific parameters. *Struct Equ*Modeling*.* 2007;14:26-47.
12. Marsh, H. W. In search of golden rules: Comment on hypothesis-testing approaches to setting cutoff values for fit indexes and dangers in overgeneralizing Hu and Bentler's (1999) findings. *Struct Equ*Modeling*.* 2004;11:320-341.
13. Nylund, K. L., Asparouhov, T. & Muthen, B. O. Deciding on the number of classes in latent class analysis and growth mixture modeling: A Monte Carlo simulation study (vol 14, pg 535, 2007). *Struct Equ*Modeling*.* 2008;15:182-182.
14. Vrieze, S. I. Model Selection and Psychological Theory: A Discussion of the Differences Between the Akaike Information Criterion (AIC) and the Bayesian Information Criterion (BIC). *Psychol Methods* 2012;17:228-243.
